# Trend and Impact of Concomitant CABG and Multiple-Valve Procedure on In-hospital Outcomes of SAVR Patients

**DOI:** 10.3389/fcvm.2021.740084

**Published:** 2021-09-03

**Authors:** Jing Wu, Xiaoqiang Cong, Zhiyang Lou, Mingyou Zhang

**Affiliations:** ^1^Institute of Translational Medicine, The First Hospital of Jilin University, Changchun, China; ^2^Department of Cardiovascular Medicine, The First Hospital of Jilin University, Changchun, China

**Keywords:** SAVR, concomitant CABG, multiple-valve procedure, in-hospital outcomes, NIS

## Abstract

**Background:** The trends of concomitant CABG and multiple-valve procedures and their impact on in-hospital outcomes in the context of transcatheter aortic valve replacement are unexplored.

**Methods:** This was a retrospective cohort study using the administrative database of the U.S. national inpatient sample from 2012 to 2018 to identify patients who underwent SAVR with or without concomitant CABG and/or multiple-valve procedures.

**Results:** During the study period, a total of 75,763 representing 378,815 patients underwent SAVR nationwide were identified, of whom 42,993 (55.1%) experienced isolated SAVR, 27,133 (34.8%) underwent concomitant CABG, 5,637 (7.2%) underwent multiple-valve procedures, and 2,298 (2.9%) underwent both concomitant CABG and multiple-valve procedures. The rate of multiple-valve procedures increased from 6.1% in 2012 to 9.2% in 2018 (*P* < 0.001 for trend). In-hospital mortality was 2.1, 3.9, 7.3, and 11.2% for isolated SAVR, SAVR with CABG, SAVR with multiple-valve procedures, and SAVR with CABG and multiple-valve procedures, respectively. After propensity matching, compared to isolated SAVR, the risk ratio for in-hospital mortality associated with concomitant CABG was 1.54 (CI 1.39-1.70). In multiple-valve procedures, it was 2.36 (CI 1.97-2.83), and in concomitant CABG and multiple-valve procedures, it was 2.92 (CI 2.29-3.73).

**Conclusions:** The proportion of patients receiving multiple-valve procedures is increasing. While concomitant CABG moderately increased in-hospital mortality, multiple-valve procedures dramatically increased in-hospital mortality and complications, even after propensity score matching

## Background

Surgical aortic valve replacement (SAVR) is still the standard care for aortic stenosis, even with the rapid expansion of transcatheter aortic valve replacement (TAVR). Furthermore, many aortic valve replacement candidates are comorbid with coronary artery disease and multiple-valve diseases. It is common practice for surgeons to perform CABG and other valve procedures in addition to aortic valve replacement for patients comorbid with coronary artery disease and other valve dysfunction during the operation. This fixation of many issues with one combined procedure has a potential advantage compared to TAVR.

Data from the Society of Thoracic Surgeons Database between 1993 and 2007 showed that 45% of patients undergoing valve surgery received concomitant CABG, and 10.9% underwent multiple-valve procedures (1). However, the advent of the TAVR era is expected to significantly change the characteristics of the SAVR population as more high operative risk patients shift to TAVR. In the background of TAVR expansion. The practice pattern for surgeons to perform isolated SAVR or combined procedures and the impact of combining CABG and/or multiple valve procedures on in-hospital outcomes have not been sufficiently investigated in the TAVR era.

## Methods

We performed a retrospective cohort study of patients undergoing surgical aortic valve replacement from the health care cost and utilization project nationwide inpatient sample (NIS) database in the United States from 2012 to 2018. NIS is the largest all-payer health care administrative database in the United States. It contains over 7 million unweighted hospital admissions each year, representing ~20% of the total annual hospital discharge in the United States. The NIS has patient-level and hospital-level data, including demographic and clinical characteristics and discharge and cost information. NIS reports data using the International Classification of Diseases-9th Revision (ICD-9) to September 2015, while data from October 2015 to 2018 are reported utilizing the International Classification of Diseases-10th Revision (ICD-10) codes. The NIS dataset used in the study contains publicly available de-identified data; therefore, this study was exempt from Institutional Review Board evaluation. Data Use Agreement were signed and complied.

**Figure 1:**
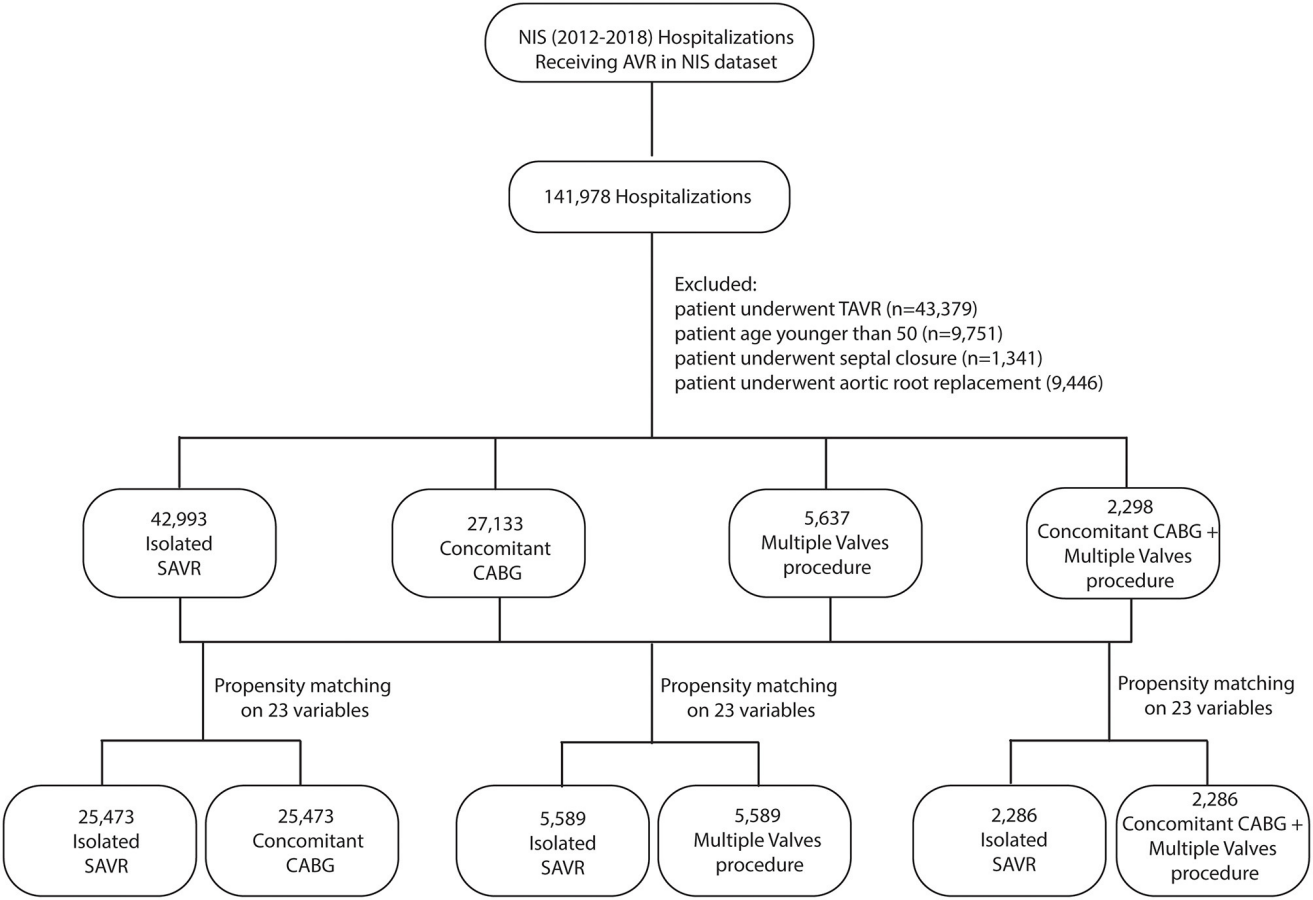
Study flow chart. NIS, national inpatient sample; CABG, coronary artery bypass grafting; SAVR, surgical aortic valve replacement; TAVR, transcatheter aortic valve replacement.

The study flowchart is outlined in Figure 1. The NIS database from 2012 to 2018 was queried to identify hospital admissions for patients undergoing aortic valve replacement. The ICD-9 and ICD-10 codes used to identify clinical characteristics and inpatient outcomes are listed in Supplementary Table 1.

Our main aim was to compare the temporal trends and outcomes of SAVR with or without concomitant CABG and/or additional valve procedures. The primary study outcome was in-hospital mortality. Other outcomes included acute stroke, acute kidney injury, new dialysis, permanent pacemaker implantation, blood transfusion, cardiac complications, tamponade, acquired pneumonia, urinary tract infection, sepsis, over 96 h of mechanical ventilation, tracheostomy, gastrostomy, cardiac arrest, cardiac shock, discharge to a nursing facility, length of hospital stay, and medical cost.

We employed propensity score methodology to match hospitalized patients undergoing isolated SAVR to those undergoing SAVR with concomitant CABG. These are also matched to those undergoing multiple-valve procedures and those undergoing concomitant CABG and multiple-valve procedures at a 1:1 ratio. Matching was performed using the MatchIt R package (2). The nearest neighbor technique was adopted to match each case to the control, which was closest in terms of the calculated propensity score, with a caliper width of 0.1. The propensity score was calculated from the following 23 matching variables: age, sex, elective admission, insurance status, presence of atrial fibrillation, hypertension, diabetes mellitus, diabetes with chronic complications, congestive heart failure, chronic lung disease, chronic renal disease, chronic anemia, chronic arthritis, coagulopathy, hypothyroidism, chronic liver disease, obesity, weight loss, peripheral artery disease, chronic pulmonary circulatory disorder, tumor, hospital bed size, and hospital teaching status. The NIS data were merged with cost-to-charge ratios available from the Healthcare Cost and Utilization Project to estimate the cost of hospitalization. We estimated the cost of stay of each inpatient by multiplying the total hospital charge with cost-to-charge ratios. We conducted a multivariable analysis to identify clinical and hospital characteristics that independently predict in-hospital mortality. A cohort after propensity matching was used for the subgroup analysis to maintain the balance between isolated SAVR and those with concomitant CABG and/or multiple-valve procedures. Estimation of the U.S. national hospitalization population is conducted by using standardized sampling and weighting methods provided by the agency for healthcare research and quality (3). We report categorical variables as proportions, while we report continuous variables as the mean ± SD or median [interquartile range (IQR)] whenever appropriate. We compared categorical values using the chi-square test and continuous variables using the Student's *t*-test. We calculated temporal trends using the Cochran-Armitage trend test. We used the Breslow-Day test to measure the homogeneity of the odds ratio (OR). We used OR and 95% confidence interval (CI) to express effect sizes. Associations were considered significant if the *p*-value was < 0.05. All statistical analyses were performed with SAS 9.4 (SAS Institute Inc., Cary, North Carolina) and R software version 3.5.2 (R Foundation for Statistical Computing, Vienna).

## Results

**Figure 2:**
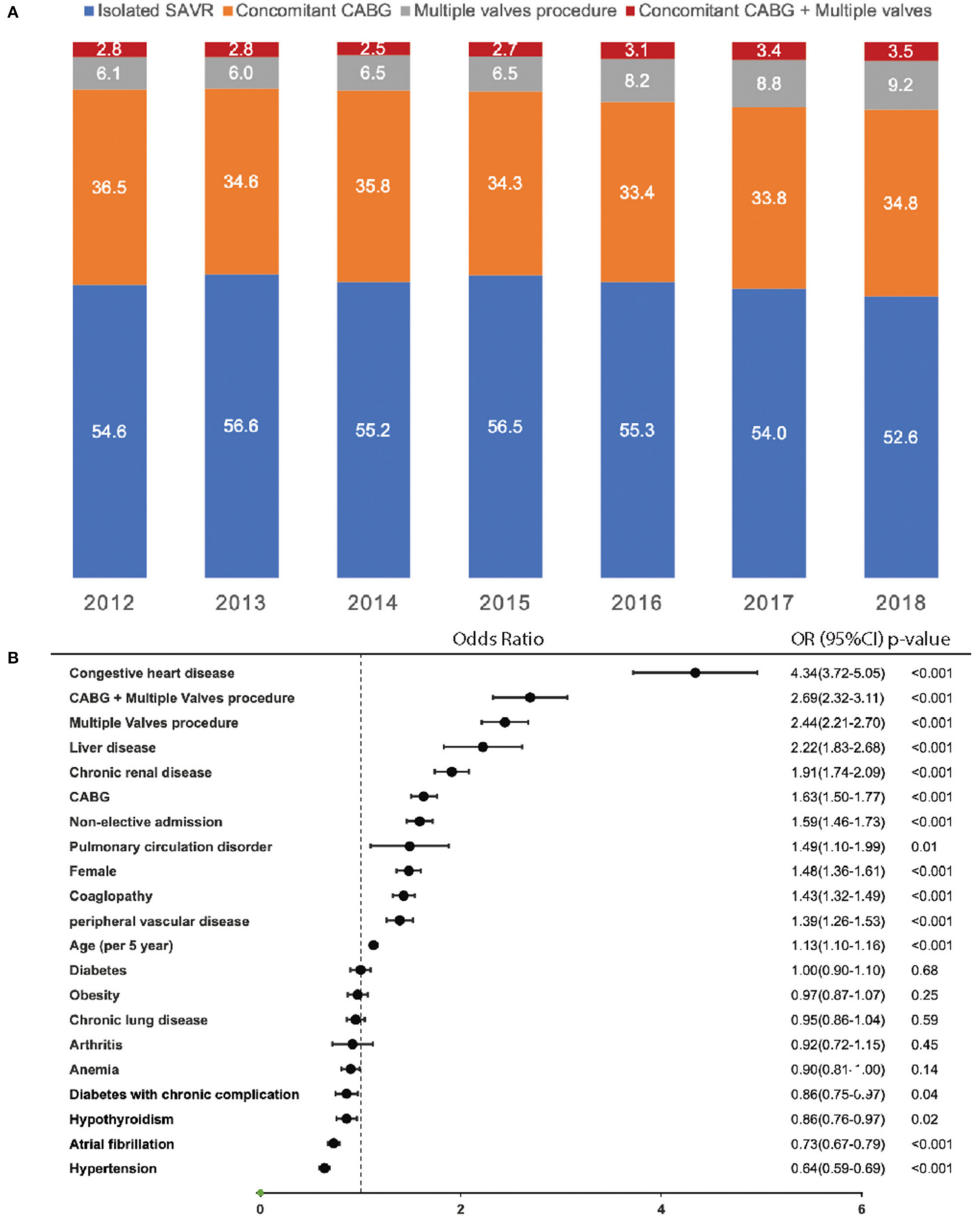
Trend of concomitant CABG and multiple valve procedures in SAVR and predictors of in-hospital mortality. **(A)** Percentage of different types of procedures in SAVR from 2012 to 2018; **(B)** predictor for in-hospital mortality. CABG, coronary artery bypass grafting; SAVR, surgical aortic valve replacement.

A total of 141,978 unweighted hospitalizations representing 709,890 hospitalizations received aortic valve replacement (AVR) nationwide in the United States between 2012 and 2018 was identified, after excluding those undergoing TAVR (*n* = 43,379), those younger than 50 (*n* = 9,751), those undergoing atrial or ventricular septal closure (*n* = 1,341), and those undergoing aortic root replacement (*n* = 9,446). The final cohort, including 78,161 unweighted hospitalizations, consisted of 42,993 (55.1%) isolated SAVR, 27,133 (34.8%) SAVR with concomitant CABG, 5,637 (7.2%) SAVR with other valve procedures, and 2,298 (2.9%) SAVR with both concomitant CABG and other valve procedures. The rate of multiple-valve procedures increased from 6.1% in 2012 to 9.2% in 2018 (*P* < 0.001 for trend). The rate of CABG plus multiple-valve procedures increased from 2.8% in 2012 to 3.5% in 2018 (*P* < 0.001 for trend) (Figure 2A).

In the final cohort, the baseline patient characteristics of those undergoing isolated SAVR or concomitant procedures are shown in Table 1. Compared to isolated SAVR, patients with concomitant CABG are older, while patients with multiple-valve procedures are slightly younger. More males underwent concomitant CABG, while more females underwent multiple-valve procedures. More patients underwent concomitant CABG comorbid with hypertension and diabetes, while fewer patients comorbid with hypertension and diabetes in patients underwent multiple-valve procedures. More patients receive multiple-valve procedures comorbid with chronic lung disease, congestive heart failure, atrial fibrillation, chronic renal disease, anemia, arthritis, coagulopathy, liver disease, weight loss, and pulmonary circulation disorder. Patients receiving multiple-valve procedures are more likely to be admitted to a large teaching hospital and more likely to be admitted as emergency cases.

**Table 1:** Baseline Characteristics for SAVR with or without concomitant CABG and/or multiple-valves procedure in the unmatched cohorts.

	**Isolated SAVR**	**Concomitant CABG**	**Multiple valves procedure**	**Concomitant CABG + multiple valves procedure**	***P*-value concomitant CABG vs. isolated SAVR**	***P*-value multiple valves procedure vs. isolated SAVR**	***P*-valueconcomitant CABG + multiple valves vs. isolated SAVR**
	**(*N* = 42,993)**	**(*N* = 27,133)**	**(*N* = 5,637)**	**(*N* = 2,298)**			
Age, yrs.	69.5 ± 9.4	72.4 ± 8.5	69.0 ± 9.4	71.8 ± 8.8	<0.001	<0.001	<0.001
Female	16,854 (39.2)	7,280 (26.8)	2,669 (47.4)	857 (37.3)	<0.001	<0.001	0.07
Race					<0.001	<0.001	<0.001
White	33,885 (83.5)	22,233 (86.6)	4,048 (75.9)	1,811 (83.7)			
Black	2,080 (5.1)	923 (3.6)	515 (9.7)	115 (5.3)			
Hispanic	2,747 (6.8)	1,377 (5.4)	370 (6.9)	130 (6.0)			
Hypertension	30,054 (69.9)	19,620 (72.3)	641 (56.9)	1,380 (60.1)	<0.001	<0.001	<0.001
Diabetes	9,281 (21.6)	6,961 (25.7)	892 (15.8)	417 (18.1)	<0.001	<0.001	<0.001
Diabetes with chronic complications	3,997 (9.3)	3,789 (14.0)	615 (10.9)	334 (14.5)	<0.001	<0.001	<0.001
Chronic lung disease	9,209 (21.4)	5,947 (21.9)	1,371 (24.3)	567 (24.7)	0.12	<0.001	<0.001
Congestive heart failure	618 (1.4)	485 (1.8)	325 (5.8)	142 (6.2)	<0.001	<0.001	<0.001
Atrial fibrillation	19,610 (45.6)	13,053 (48.1)	3,578 (63.5)	1,380 (60.1)	<0.001	<0.001	<0.001
Chronic renal disease	6,424 (14.9)	5,429 (20.0)	1,319 (23.4)	643 (28.0)	<0.001	<0.001	<0.001
Anemia	6,841 (15.9)	4,534 (16.7)	1,176 (20.9)	418 (18.2)	<0.001	<0.001	0.004
Arthritis	1,385 (3.2)	755 (2.8)	221 (3.9)	70 (3.0)	0.001	0.001	0.69
Coagulopathy	14,342 (33.4)	10,107 (37.2)	2,574 (45.7)	1,084 (47.2)	<0.001	<0.001	<0.001
Hypothyroidism	6,036 (14.0)	3,479 (12.8)	857 (15.2)	341 (14.8)	<0.001	0.02	0.30
Liver disease	960 (2.2)	483 (1.8)	232 (4.1)	70 (3.0)	<0.001	<0.001	0.01
Obesity	10,538 (24.5)	6,122 (22.6)	1,095 (19.4)	381 (16.6)	<0.001	<0.001	<0.001
Weight loss	1,629 (3.8)	1,322 (4.9)	635 (11.3)	256 (11.1)	<0.001	<0.001	<0.001
Peripheral vascular disease	6,582 (15.3)	5,082 (18.7)	835 (14.8)	447 (19.5)	<0.001	<0.001	<0.001
Pulmonary circulation disorder	151 (0.3)	110 (0.4)	72 (1.3)	35 (1.5)	0.28	<0.001	<0.001
Tumor	489 (1.1)	368 (1.4)	68 (1.2)	28 (1.2)	0.003	0.70	0.80
Teaching hospital	33,862 (78.8)	20,559 (75.8)	4,703 (83.4)	1,860 (80.9)	<0.001	<0.001	<0.001
Rural location	935 (2.2)	711 (2.6)	72 (1.3)	37 (1.6)	0.14	<0.001	<0.001
Large hospital bed size	29,907 (69.6)	18,391 (67.8)	4,111 (73.5)	1,587 (69.1)	<0.001	<0.001	0.87
**Primary payer**							
Medicare/medicaid	29,972 (69.7)	21,276 (78.4)	6,492 (73.0)	1,850 (80.6)	<0.001	<0.001	<0.001
Private insurance	11,457 (26.7)	4,998 (18.4)	1,285 (22.8)	370 (16.1)	<0.001	<0.001	<0.001
Elective admission	33,321 (77.7)	18,317 (67.8)	3,550 (63.2)	1,387 (60.6)	<0.001	<0.001	<0.001
0-25th percentile income	10,782 (25.6)	6,045 (22.7)	1,385 (25.1)	473 (21.1)	<0.001	0.007	<0.001

*Values are count (percent), mean ± SD, median (interquartile range). SAVR, surgical aortic valve replacement; CABG, coronary artery bypass grafting*.

Before propensity matching, in-hospital mortality was 2.1, 3.9, 7.3, and 11.2% in isolated SAVR, concomitant CABG, multiple-valve procedures, and concomitant CABG and multiple-valve procedures, respectively (Table 2). The rate of stroke, acute kidney injury, new dialysis, cardiac complications, acquired pneumonia, urinary tract infection, sepsis, mechanical ventilation, tracheostomy, gastrostomy, cardiac arrest, and cardiac shock complications are all increased with concomitant CABG and/or multiple-valve procedures. In addition to the length of hospital stays and cost. The annual rates of in-hospital mortality, stroke, acute kidney injury, cardiac complications, cardiac arrest, shock, and individual hospitalization costs in each calendar year during the study period are shown in Supplementary Figures 1–7.

**Table 2:** In hospital outcomes of SAVR with or without concomitant CABG and/or multiple valves procedure in the unmatched cohorts.

	**Isolated SAVR**	**Concomitant CABG**	**Multiple valves procedure**	**Concomitant CABG + multiple valves procedure**	***P*-value concomitant CABG vs. isolated SAVR**	***P*-valueMultiple valves procedure vs. isolated SAVR**	***P*-value Concomitant CABG + multiple valves vs. isolated SAVR**
	**(*N* = 42,993)**	**(*N* = 27,133)**	**(*N* = 5,637)**	**(*N* = 2,298)**			
In-hospital death	906 (2.1)	1,056 (3.9)	410 (7.3)	258 (11.2)	<0.001	<0.001	<0.001
Stroke	1,120 (2.6)	856 (3.2)	283 (5.0)	124 (5.4)	<0.001	<0.001	<0.001
Acute kidney injury	7,058 (16.4)	6,455 (23.8)	1,868 (33.1)	941 (40.9)	<0.001	<0.001	<0.001
New dialysis	586 (1.4)	497 (1.8)	229 (4.1)	122 (5.3)	<0.001	<0.001	<0.001
Pacemaker implantation	2,399 (5.6)	1,521 (5.6)	766 (13.6)	270 (11.8)	0.90	<0.001	<0.001
Blood transfusion	10,609 (24.7)	8,401 (31.0)	1,754 (31.1)	766 (33.3)	<0.001	<0.001	<0.001
Cardiac complications	5,412 (12.6)	3,644 (13.4)	863 (15.3)	346 (15.1)	0.002	<0.001	<0.001
Tamponade	452 (1.1)	288 (1.1)	117 (2.1)	43 (1.9)	0.93	<0.001	<0.001
Acquired pneumonia	883 (2.1)	823 (3.0)	253 (4.5)	158 (6.9)	<0.001	<0.001	<0.001
Urinary tract infection	2,180 (5.1)	1,617 (6.0)	512 (9.1)	222 (9.7)	<0.001	<0.001	<0.001
Sepsis	1,117 (2.6)	796 (2.9)	434 (7.7)	178 (7.7)	0.008	<0.001	<0.001
Mechanical ventilation	1,271 (3.0)	1,209 (4.5)	547 (9.7)	285 (12.4)	<0.001	<0.001	<0.001
Tracheostomy	551 (1.3)	492 (1.8)	245 (4.3)	144 (6.3)	<0.001	<0.001	<0.001
Gastrostomy	377 (0.9)	368 (1.4)	119 (2.1)	79 (3.4)	<0.001	<0.001	<0.001
Cardiac arrest	945 (2.2)	972 (3.6)	224 (4.0)	142 (6.2)	<0.001	<0.001	<0.001
Cardiac shock	1,558 (3.6)	1,619 (6.0)	654 (11.6)	343 (14.9)	<0.001	<0.001	<0.001
Discharge status					<0.001	<0.001	<0.001
Routine	15,707 (36.6)	7,607 (28.1)	1,348 (23.9)	394 (17.2)			
Home health care	9,883 (23.0)	8,990 (33.2)	1,940 (34.4)	948 (41.3)			
Other care facility	16,165 (37.6)	9,198 (33.9)	1,846 (32.8)	653 (28.5)			
Length of stay, days	7 (5, 10)	8 (6, 13)	11 (7, 17)	12 (8, 20)	<0.001	<0.001	<0.001
Mean cost, $	48,371 ± 35,313	56,935 ± 36,325	79,961 ± 60,544	88,770 ± 66,160	<0.001	<0.001	<0.001

*Values are count (percent), mean ± SD, median (interquartile range). SAVR, surgical aortic valve replacement; CABG, coronary artery bypass grafting*.

The baseline characteristics comparing those undergoing concomitant CABG, those undergoing multiple-valve procedures, and those undergoing concomitant CABG and multiple-valve procedures to those undergoing isolated SAVR after propensity matching are shown in Supplementary Tables 2–4. After matching, the standardized differences were <10% for all characteristics compared to isolated SAVR.

### SAVR With Concomitant CABG vs. Isolated SAVR

After propensity matching, in-hospital outcomes compared to patients undergoing SAVR with concomitant CABG to isolated CABG are shown in Table 3. Those receiving concomitant CABG had higher in-hospital mortality [3.8 vs. 2.5%; odds ratio (OR): 1.54; 95% confidence interval (CI): 1.39-1.70; *p* <0.001], acute kidney injury (22.7 vs. 19.7%; OR: 1.20; 95% CI: 1.15-1.25; *p* <0.001), blood transfusion (31.1 vs. 26.0%; OR: 1.28; 95% CI: 1.23-1.32; *p* <0.001), cardiac complications (13.7 vs. 12.9%; OR: 1.08; 95% CI: 1.02-1.13; *p* = 0.005), acquired pneumonia (3.0 vs. 2.4%; OR: 1.27; 95% CI: 1.14-1.42; *p* <0.001), mechanical ventilation (4.4 vs. 3.4%; OR: 1.29; 95% CI: 1.18-1.41; *p* <0.001), cardiac arrest (3.5 vs. 2.5%; OR: 1.45; 95% CI: 1.30-1.60; *p* <0.001), and cardiac shock (5.7 vs. 4.0%; OR: 1.44; 95% CI: 1.33-1.56; *p* <0.001); longer lengths of hospital stay [median 8 days (IQR: 6-12 days) vs. median 7 days (IQR: 5-11 days); *P* <0.001]; higher hospital costs ($56,935 ± 36,325 vs. $48,371 ± 35,313); and lower rate of routine discharge (28.5 vs. 33.3%; OR:0.80; 95% CI: 0.77-0.83; *p* <0.001) than those who undergoing isolated SAVR. No difference was observed in the rate of stroke, new dialysis, tamponade, urinary tract infection, tracheostomy, and gastrostomy between patient undergoing SAVR with and without concomitant CABG.

**Table 3:** In-hospital Outcomes for isolated SAVR and SAVR with concomitant CABG in the Propensity Matched Cohorts.

	**Isolated SAVR**	**Concomitant CABG**	***P*-value**
	**(*N* = 25,473)**	**(*N* = 25,473)**	
In-hospital death	636 (2.5)	966 (3.8)	<0.001
Stroke	782 (3.1)	770 (3.0)	0.78
Acute kidney injury	5,014 (19.7)	5,780 (22.7)	<0.001
New dialysis	426 (1.7)	441 (1.7)	0.63
Pacemaker implantation	1,570 (6.1)	1,389 (5.5)	0.001
Blood transfusion	6,630 (26.0)	7,911 (31.1)	<0.001
Cardiac complications	3,282 (12.9)	3,498 (13.7)	0.005
Tamponade	299 (1.1)	272 (1.1)	0.27
Acquired pneumonia	614 (2.4)	776 (3.0)	<0.001
Urinary tract infection	1,446 (5.7)	1,522 (6.0)	0.16
Sepsis	800 (3.1)	730 (2.9)	0.07
Mechanical ventilation	873 (3.4)	1,113 (4.4)	<0.001
tracheostomy	399 (1.6)	452 (1.8)	0.07
Gastrostomy	279 (1.1)	325 (1.3)	0.07
Cardiac arrest	631 (2.5)	902 (3.5)	<0.001
Cardiac shock	1,030 (4.0)	1,456 (5.7)	<0.001
**Discharge status**			
Routine	8,488 (33.3)	7,266 (28.5)	<0.001
Home health care	6,826 (26.8)	8,273 (32.5)	<0.001
Other care facility	9,301 (36.5)	8,713 (34.2)	<0.001
Length of stay, days	7 (5, 11)	8 (6, 12)	<0.001
Mean cost, $	50,609 ± 37,080	56,083 ± 35,438	<0.001

*Values are count (percent), mean ± SD, median (interquartile range). SAVR, surgical aortic valve replacement; CABG, coronary artery bypass grafting*.

### Multiple-Valve Procedure vs. Isolated SAVR

After propensity matching, in-hospital outcomes comparing patients undergoing multiple-valve procedure to those undergoing isolated CABG are shown in Table 4. Those undergoing multiple-valve procedure had higher in-hospital mortality (7.3 vs. 3.2%; OR: 2.36; 95% CI: 1.97-2.83; *p* <0.001), stroke (5.0 vs. 3.8%; OR: 1.31; 95% CI: 1.09-1.57; *p* = 0.006), acute kidney injury (33.2 vs. 24.7%; OR: 1.51; 95% CI: 1.39-1.64; *p* <0.001), new dialysis (4.0 vs. 2.8%; OR: 1.44; 95% CI: 1.17-1.77; *p* = 0.004), pace maker implantation (13.6 vs. 6.8%; OR: 2.16; 95% CI: 1.90-2.45; *p* <0.001), blood transfusion (31.1 vs. 27.8%; OR: 1.17; 95% CI: 1.08-1.27; *p* <0.001), acquired pneumonia (4.5 vs. 3.3%; OR: 1.36; 95% CI: 1.12-1.65; *p* = 0.002), urinary tract infection (9.1 vs. 7.8%; OR: 1.36; 95% CI: 1.12-1.65; *p* = 0.02), sepsis (7.6 vs. 5.4%; OR: 1.44; 95% CI: 1.24-1.68; *p* <0.001), mechanical ventilation (9.6 vs. 6.6%; OR: 1.50; 95% CI: 1.30-1.70; *p* <0.001), and cardiac shock (11.6 vs. 6.1%; OR: 2.0; 95% CI: 1.75-2.30; *p* <0.001); longer lengths of hospital stay [median 11 days (IQR: 7-11 days) vs. median 8 days (IQR: 6-14 days); *P* <0.001]; higher hospital costs ($80,024 ± 60,468 vs. $59,548 ± 51,212); and lower rates of routine discharge (23.9 vs. 30.5%; OR: 0.71; 95% CI: 0.66-0.78; *p* <0.001) than those who underwent isolated SAVR. No difference was observed in the rates of cardiac complications, tamponade, tracheostomy, gastrostomy, or cardiac shock between patients undergoing SAVR with and without multiple-valve procedures.

**Table 4:** In-hospital Outcomes for isolated SAVR and SAVR with Multiple valves procedure in the Propensity Matched Cohorts.

	**Isolated SAVR**	**Multiple valves procedure**	***P*-value**
	**(*N* = 5,589)**	**(*N* = 5,589)**	
In-hospital death	900 (3.2)	407 (7.3)	<0.001
Stroke	217 (3.8)	280 (5.0)	0.004
Acute kidney injury	1,380 (24.7)	1,853 (33.2)	<0.001
New dialysis	158 (2.8)	225 (4.0)	<0.001
Pacemaker implantation	380 (6.8)	960 (13.6)	<0.001
Blood transfusion	1,556 (27.8)	1,740 (31.1)	<0.001
Cardiac complications	912 (16.3)	858 (15.4)	0.17
Tamponade	103 (1.8)	117 (2.1)	0.38
Acquired pneumonia	186 (3.3)	250 (4.5)	0.002
Urinary tract infection	438 (7.8)	509 (9.1)	0.02
Sepsis	303 (5.4)	427 (7.6)	<0.001
Mechanical ventilation	370 (6.6)	536 (9.6)	<0.001
tracheostomy	200 (3.6)	240 (4.3)	0.06
Gastrostomy	102 (1.8)	117 (2.1)	0.34
Cardiac arrest	187 (3.3)	222 (4.0)	0.09
Cardiac shock	343 (6.1)	647 (11.6)	<0.001
**Discharge status**			
Routine	1,704 (30.5)	1,334 (23.9)	<0.001
Home health care	1,664 (29.8)	1,918 (34.3)	<0.001
Other care facility	1,989 (35.6)	1,838 (32.9)	0.003
Length of stay, days	8 (6, 14)	11 (7, 17)	<0.001
Mean cost, $	59,548 ± 51,212	80,024 ± 60,468	<0.001

*Values are count (percent), mean ± SD, median (interquartile range). SAVR, surgical aortic valve replacement*.

### Concomitant CABG and Multiple-Valve Procedure vs. Isolated SAVR

After propensity matching, in-hospital outcomes compared to patients undergoing concomitant CABG and multiple-valve procedures, to isolated CABG are shown in Table 5. Those undergoing multiple-valves procedure had the following: higher in-hospital mortality (11.3 vs. 4.2%; OR: 2.92; 95% CI: 2.29-3.73; *p* <0.001), acute kidney injury (41.0 vs. 27.8%; OR: 1.81; 95% CI: 1.60-2.05; *p* <0.001), new dialysis (5.3 vs. 3.5%; OR: 1.54; 95% CI: 1.15-2.06; *p* = 0.004), pace maker implantation (11.8 vs. 7.0%; OR: 1.76; 95% CI: 1.43-2.16; *p* <0.001), blood transfusion (33.4 vs. 28.4%; OR: 1.26; 95% CI: 1.11-1.43; *p* <0.001), acquired pneumonia (6.8 vs. 3.5%; OR: 2.05; 95% CI: 1.55-2.70; *p* <0.001), mechanical ventilation (12.5 vs. 7.8%; OR: 1.68; 95% CI: 1.38-2.04; *p* <0.001), tracheostomy (6.3vs. 4.9%; OR: 1.30; 95% CI: 1.01-1.68; *p* = 0.05), cardiac arrest (6.2 vs. 3.3%; OR: 1.93; 95% CI: 1.45-2.56; *p* <0.001), and cardiac shock (15.0 vs. 7.1%; OR: 2.31; 95% CI: 1.89-2.81; *p* <0.001); longer lengths of hospital stay [median 12 days (IQR: 8 to 20 days) vs. median 8 days (IQR: 6-14 days); *P* <0.001]; higher hospital costs ($88,970 ± 66,223 vs. $60,501 ± 49,696); and lower rates of routine discharge (17.3 vs. 29.1%; OR:0.51; 95% CI: 0.44-0.58; *p* <0.001) than those who undergoing isolated SAVR. No difference was observed in the rate of cardiac complications, tamponade, or gastrostomy between patients undergoing isolated SAVR and those undergoing concomitant CABG and multiple-valve procedure.

**Table 5:** In-hospital Outcomes for isolated SAVR and SAVR with concomitant CABG + multiple valves procedure in the Propensity Matched Cohorts.

	**Isolated SAVR**	**Concomitant CABG + multiple valves procedure**	***P*-value**
	**(*N* = 2,286)**	**(*N* = 2,286)**	
In-hospital death	95 (4.2)	257 (11.3)	<0.001
Stroke	95 (4.2)	123 (5.4)	0.06
Acute kidney injury	635 (27.8)	938 (41.0)	<0.001
New dialysis	80 (3.5)	121 (5.3)	0.004
Pacemaker implantation	161 (7.0)	269 (11.8)	<0.001
Blood transfusion	650 (28.4)	763 (33.4)	<0.001
Cardiac complications	340 (14.9)	346 (15.1)	0.84
Tamponade	44 (1.9)	43 (1.9)	0.99
Acquired pneumonia	79 (3.5)	156 (6.8)	<0.001
Urinary tract infection	184 (8.0)	219 (9.6)	0.08
Sepsis	147 (6.4)	177 (7.7)	0.09
Mechanical ventilation	179 (7.8)	285 (12.5)	<0.001
tracheostomy	112 (4.9)	144 (6.3)	0.05
Gastrostomy	58 (2.5)	79 (3.5)	0.08
Cardiac arrest	76 (3.3)	142 (6.2)	<0.001
Cardiac shock	162 (7.1)	342 (15.0)	<0.001
**Discharge status**			
Routine	666 (29.1)	394 (17.3)	<0.001
Home health care	733 (32.1)	942 (41.3)	<0.001
Other care facility	761 (33.3)	648 (28.4)	<0.001
Length of stay, days	8 (6, 14)	12 (8, 20)	<0.001
Mean cost, $	60,501 ± 49,696	88,970 ± 66,223	<0.001

*Values are count (percent), mean ± SD, median (interquartile range). SAVR, surgical aortic valve replacement; CABG, coronary artery bypass grafting*.

### Predictor of the In-hospital Mortality

Using the final cohort including 78,061 hospitalizations consisting of patients with isolated SAVR and those with concomitant CABG and/or multiple-valve procedures, multivariate logistic regression was conducted to predict in-hospital mortality. Variables included in the adjusted regression model included age, sex, atrial fibrillation, elective admission, hypertension, diabetes mellitus, diabetes with chronic complications, congestive heart failure, chronic lung disease, chronic renal disease, anemia, chronic arthritis, coagulopathy, hypothyroidism, chronic liver disease, obesity, peripheral artery disease, chronic pulmonary circulatory disorder, concomitant CABG, multiple-valve procedure, and CABG with multiple-valve procedure. Factors associated with highest in hospital mortality was congestive heart failure, (OR: 4.34; 95% CI: 3.72-5.05; *p* <0.001), followed by undergoing CABG and multiple-valve procedure (OR: 2.69; 95% CI: 2.32-3.11; *p* <0.001), multiple-valves procedure (OR: 2.44; 95% CI: 2.21-2.70; *p* <0.001), liver disease (OR:2.22; 95% CI: 1.83-2.68; *p* <0.001), concomitant CABG (OR: 1.63; 95% CI: 1.50-1.77; *p* <0.001), non-elective admission (OR: 1.59; 95% CI: 1.46-1.73; *p* <0.001), pulmonary circulation disorder (OR: 1.49; 95% CI: 1.10-1.99; *p* = 0.01), female (OR:1.48; 95% CI: 1.36-1.61; *p* <0.001), coagulopathy (OR: 1.43; 95% CI: 1.32-1.56; *p* <0.001), peripheral vascular disease (OR: 1.39; 95% CI: 1.26-1.53; *p* <0.001), older age (per 5 years, OR: 1.13; 95% CI: 1.10-1.16; *p* <0.001). However, hypertension (OR: 0.64; 95% CI: 0.59-0.69; *p* <0.001) and atrial fibrillation (OR: 0.73; 95% CI: 0.67-0.79; *p* <0.001) were associated with lower in-hospital mortality (Figure 2B).

## Discussion

We described the trend of the concomitant CABG and/or multiple-valve procedure and compared the in-hospital outcomes among them in the background of TAVR expansion. The primary findings were as follows: (1) the percentage of patients undergoing multiple-valve procedures and concomitant CABG plus multiple-valve procedures increased during the study period. (2). Concomitant CABG and/or multiple-valve procedures were associated with worse in-hospital outcomes, including higher in-hospital mortality and more medical resource usage. (3) The factor associated with the highest in-hospital mortality was congestive heart failure, followed by undergoing both concomitant CABG and multiple-valve procedures, undergoing multiple-valve procedures, liver disease, chronic renal disease, and concomitant CABG.

The advent of the TAVR era dramatically changed the way clinicians manage patients needing aortic valve replacement. However, an increasing number of patients undergoing TAVR are not followed by a proportional decrease in patients undergoing SAVR. Data from this study showed that the annual number of patients undergoing SAVR remains relatively stable. The proportion of patients undergoing concomitant CABG did not increase. The proportion of patients undergoing multiple-valve procedures increased during the study period, as did the proportion of patients undergoing concomitant CABG with multiple-valve procedures.

The results from this large cohort analysis provided clear evidence that concomitant CABG in addition to SAVR was associated with the worse in-hospital outcomes, including mortality. The in-hospital mortality increase was evident even after propensity matching, which helps clarify the conflicting data regarding the impact of additional CABG on aortic valve replacement on early outcomes (4–6). Approximately 1/3 of the patients undergoing SAVR are treated concomitantly with CABG. The increased in-hospital mortality, and other worse in-hospital outcomes, are likely explained by this subset of patients being sicker, even after propensity matching, since additional coronary artery disease is an intrinsic risk for SAVR candidates. Furthermore, the additional procedure increases procedure complexity and prolongs the operation and anesthesia duration. These factors are all attributable to the worse in-hospital outcome observed in this study. Arguably, patients may benefit from treating obstructive coronary artery disease; however, this could still be controversial, as most of these coronary artery diseases are stable. Evidence from the COURAGE and ISCHEMIA trials have shown that patients with stable coronary artery disease may not benefit from invasive revascularization therapy, including CABG (7, 8). An additional procedure exposed them to additional risk for worse in-hospital outcomes. The ESC/EACTS guidelines recommended performing concomitant CABG at the time of SAVR if critical coronary stenosis was present (9). However, significant in-hospital mortality increases identified in this large cohort study still remind the clinician to individualize the decision. This is especially the case when the obstructive coronary disease was borderline or only supplying a limited amount of myocardium, performing isolated SAVR instead of combined surgery would be easier for the surgeon and more beneficial for these patients.

Multiple-valve deficits are common among patients who are candidates for aortic valve replacement. This subgroup is underrepresented in clinical trials, as they are often excluded from enrollment. Limited data exist in the literature regarding the practice pattern and optimal management for aortic replacement candidates comorbid with other valve diseases. Current analyses have shown that in real-world practice, multiple-valve procedures are increasingly performed during the study period. An STS dataset between 1993 and 2007 showed that 10.7% of patients underwent valve procedures, also underwent multiple-valve procedures. Overall, the operative mortality doubled compared with single valve procedures (10), although a decrease in mortality was observed from 1993 to 2007 (1). However, our current analysis provides clear evidence that, even with the latest advances and improvements in surgical techniques and skills, additional valve procedure still dramatically increased in-hospital mortality. Studies indicated that at the price of increased perioperative risk, late improvement in survival and clinical benefit can be expected with multiple-valve procedures (10, 11). Nevertheless, the dramatic increase in in-hospital mortality should be carefully balanced, and decisions should be made on a case-by-case basis.

## Conclusion

The percentages of SAVR patients undergoing multiple-valve procedures and multiple-valve procedures plus CABG in the United States were increased during the study period. Concomitant CABG and/or multiple-valve procedures were associated with worse in-hospital outcomes, including increased in-hospital mortality. Further studies to explore the best practice pattern for aortic valve replacement candidates who comorbid with coronary artery disease and/or other valve deficit were warranted.

### Limitations

The administrative database lacks certain clinically relevant information, such as echocardiography, laboratory and medication data, and operative information, such as procedure duration and anesthesia time. Therefore, these variables cannot be incorporated into the propensity matching. Furthermore, there are intrinsic factors associated with patients comorbid with coronary artery disease and/or other valve diseases treated with concomitant CABG and/or other valve procedures. Many large inpatient cohorts, such as NIS, are subject to coding and documentation errors. Additionally, retrospective observational analysis was liable to selection bias; however, the database has been validated internally and externally (12–14). This national dataset analysis shows the latest real-world data of combined procedure practice patterns for SAVR and their impact on in-hospital outcomes.

## Data Availability Statement

The NIS is the largest publicly available all-payer inpatient care database in the United States, governed by its Data Use Agreement, data sharing is restricted and redistribution is prohibited. Please refer to AHRQ HCUP (www.hcup-us.ahrq.gov) for more information.

## Ethics Statement

Ethical review and approval was not required for the study on human participants in accordance with the local legislation and institutional requirements. Written informed consent for participation was not required for this study in accordance with the national legislation and the institutional requirements.

## Author Contributions

MZ and JW conceived the research project and directed the analysis. MZ, JW, XC, and ZL performed data analysis. MZ and JW wrote the paper. All authors edited and approved the paper.

## Conflict of Interest

The authors declare that the research was conducted in the absence of any commercial or financial relationships that could be construed as a potential conflict of interest.

## Publisher's Note

All claims expressed in this article are solely those of the authors and do not necessarily represent those of their affiliated organizations, or those of the publisher, the editors and the reviewers. Any product that may be evaluated in this article, or claim that may be made by its manufacturer, is not guaranteed or endorsed by the publisher.

## Abbreviations

CABG: coronary artery bypass grafting
CI: confidence interval
NIS: nationwide inpatient sample
OR: odds ratio
SAVR: surgical aortic valve replacement
TAVR: transcatheter aortic valve replacement.
