# Supplementary figures and images for: Trend and Impact of Concomitant CABG and Multiple-Valve Procedure on In-hospital Outcomes of SAVR Patients
Source: Front Cardiovasc Med. 2021 Sep 3;8:740084. doi: 10.3389/fcvm.2021.740084 (PMC8446624; doi:10.3389/fcvm.2021.740084)

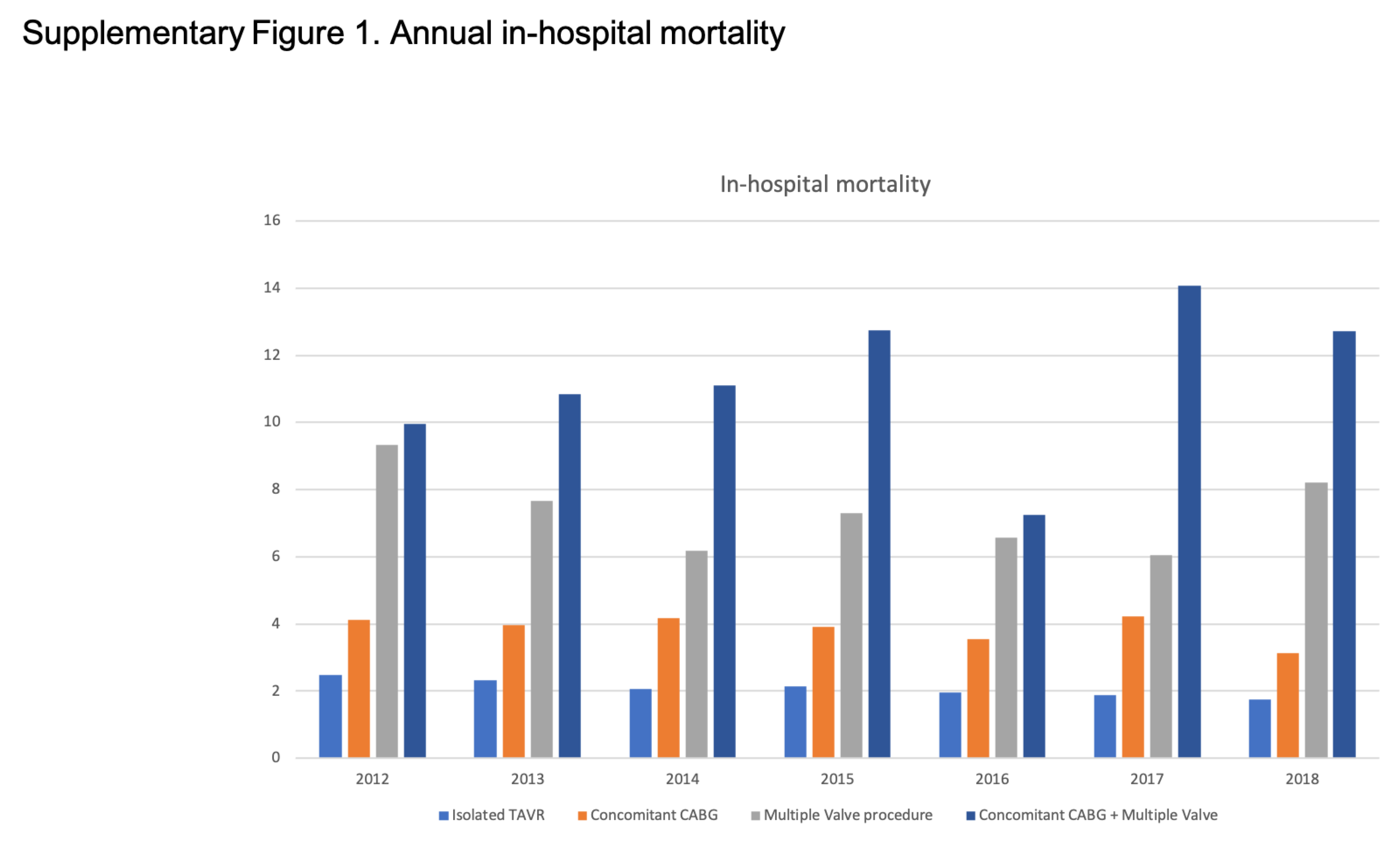

Supplement: Supplementary file 1 [file Image_1.TIF]

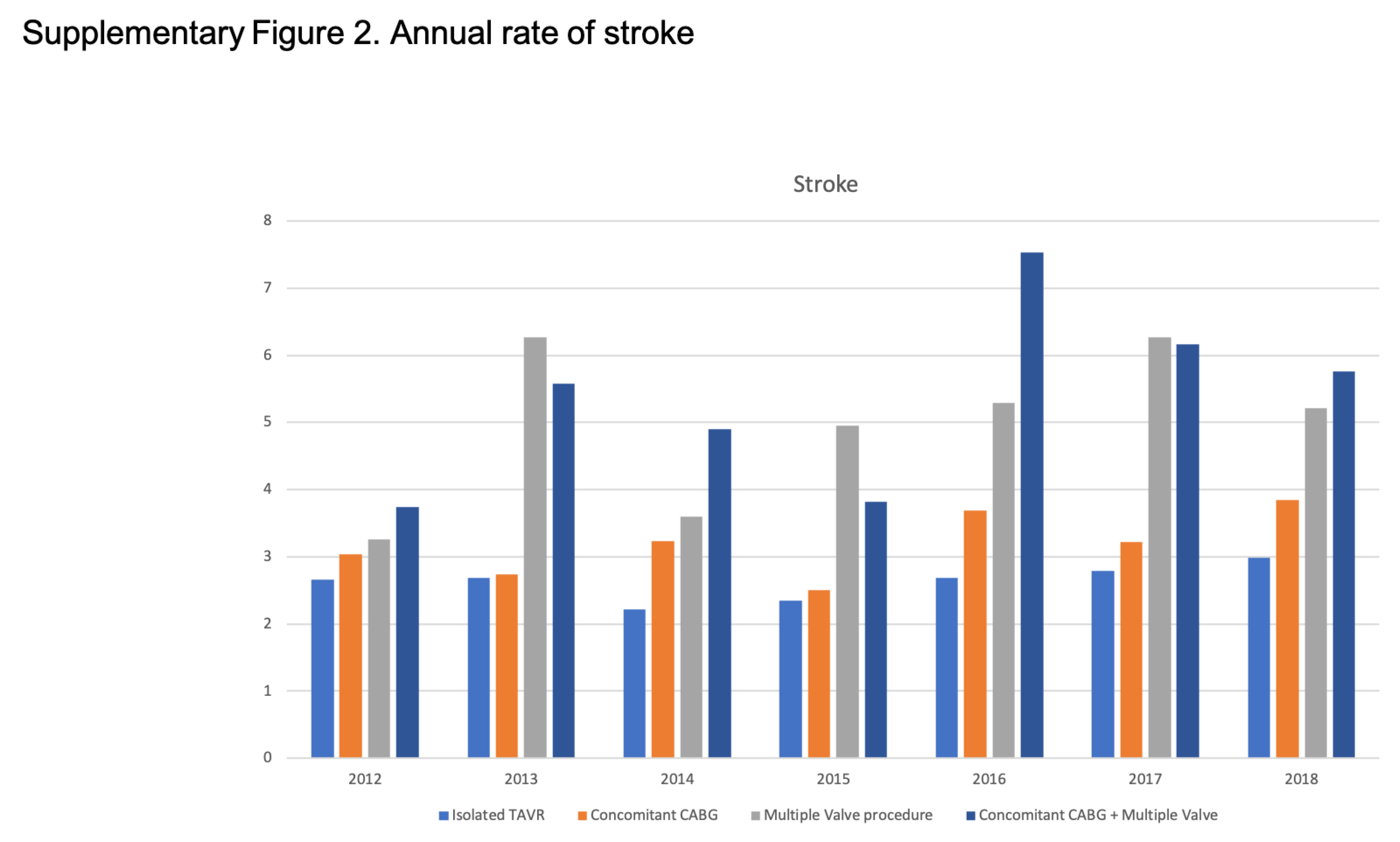

Supplement: Supplementary file 2 [file Image_2.TIF]

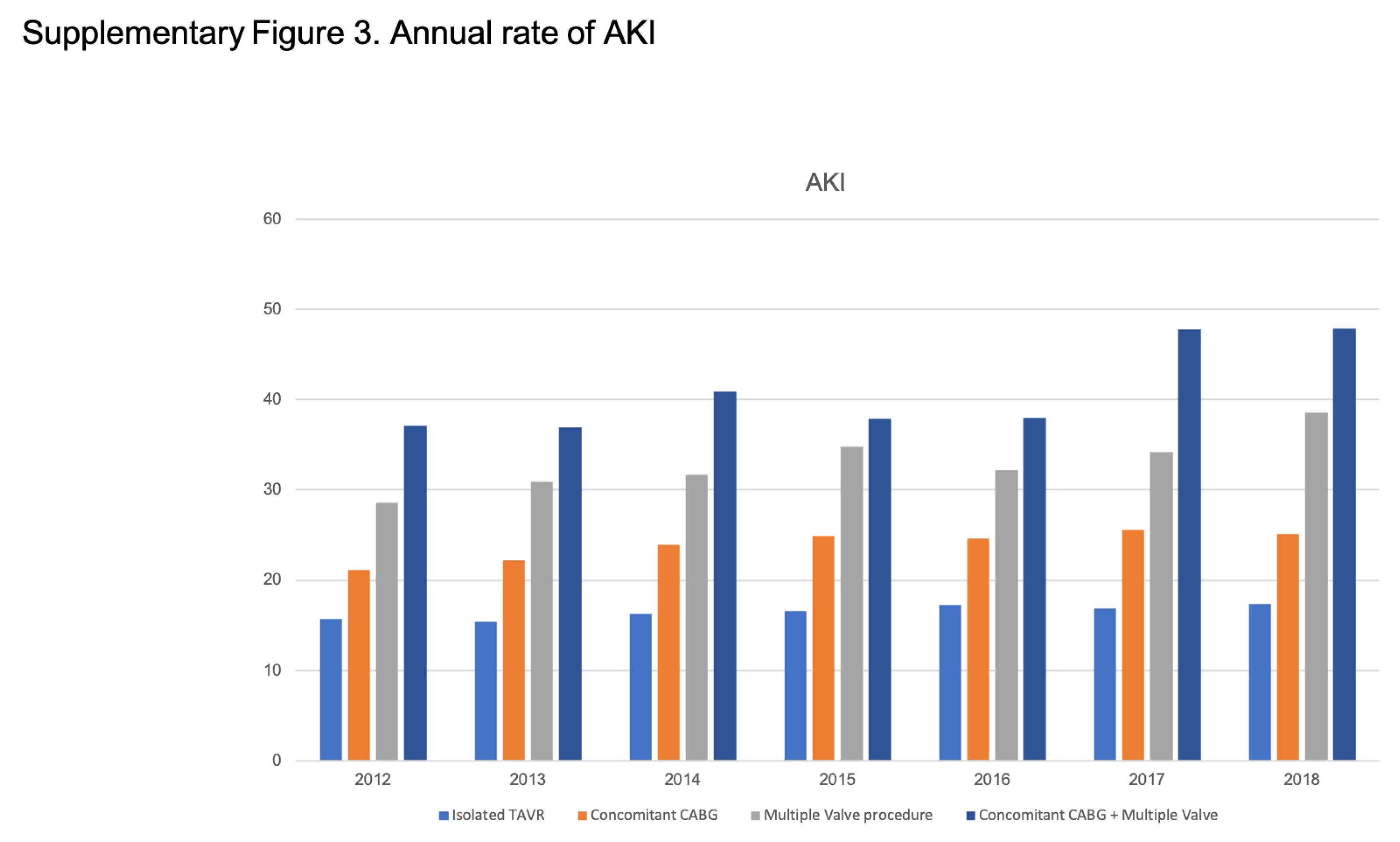

Supplement: Supplementary file 3 [file Image_3.TIF]

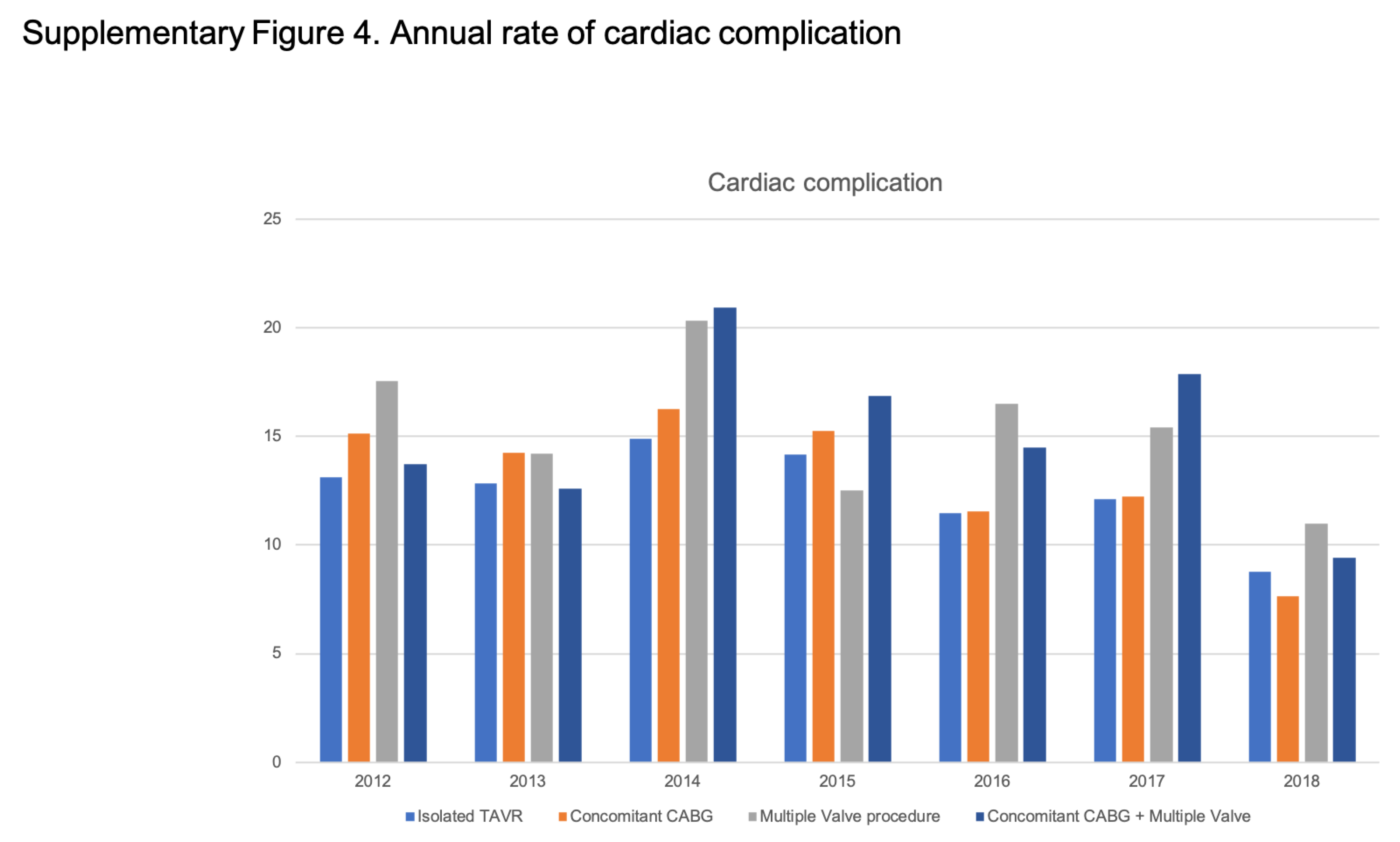

Supplement: Supplementary file 4 [file Image_4.TIF]

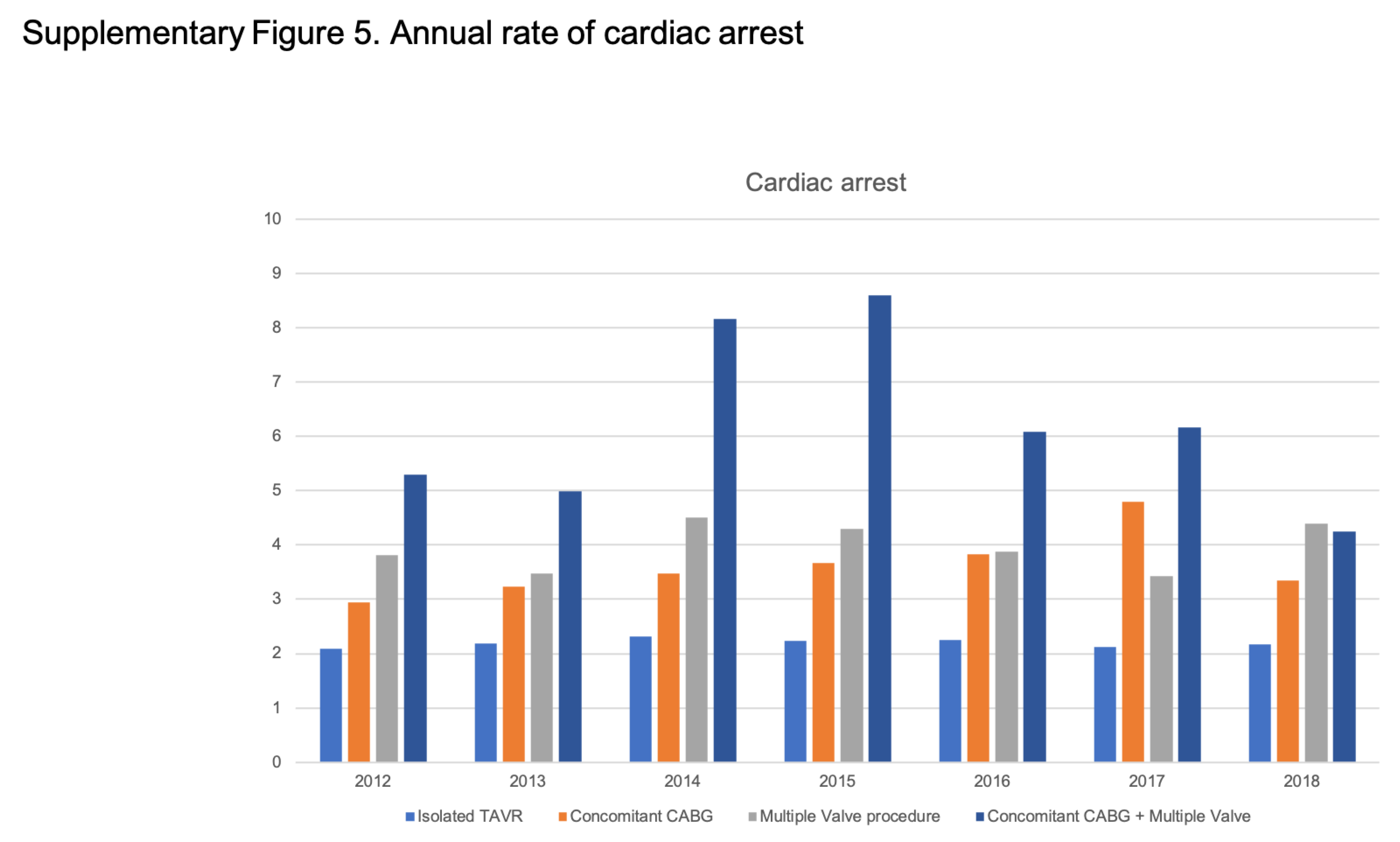

Supplement: Supplementary file 5 [file Image_5.TIF]

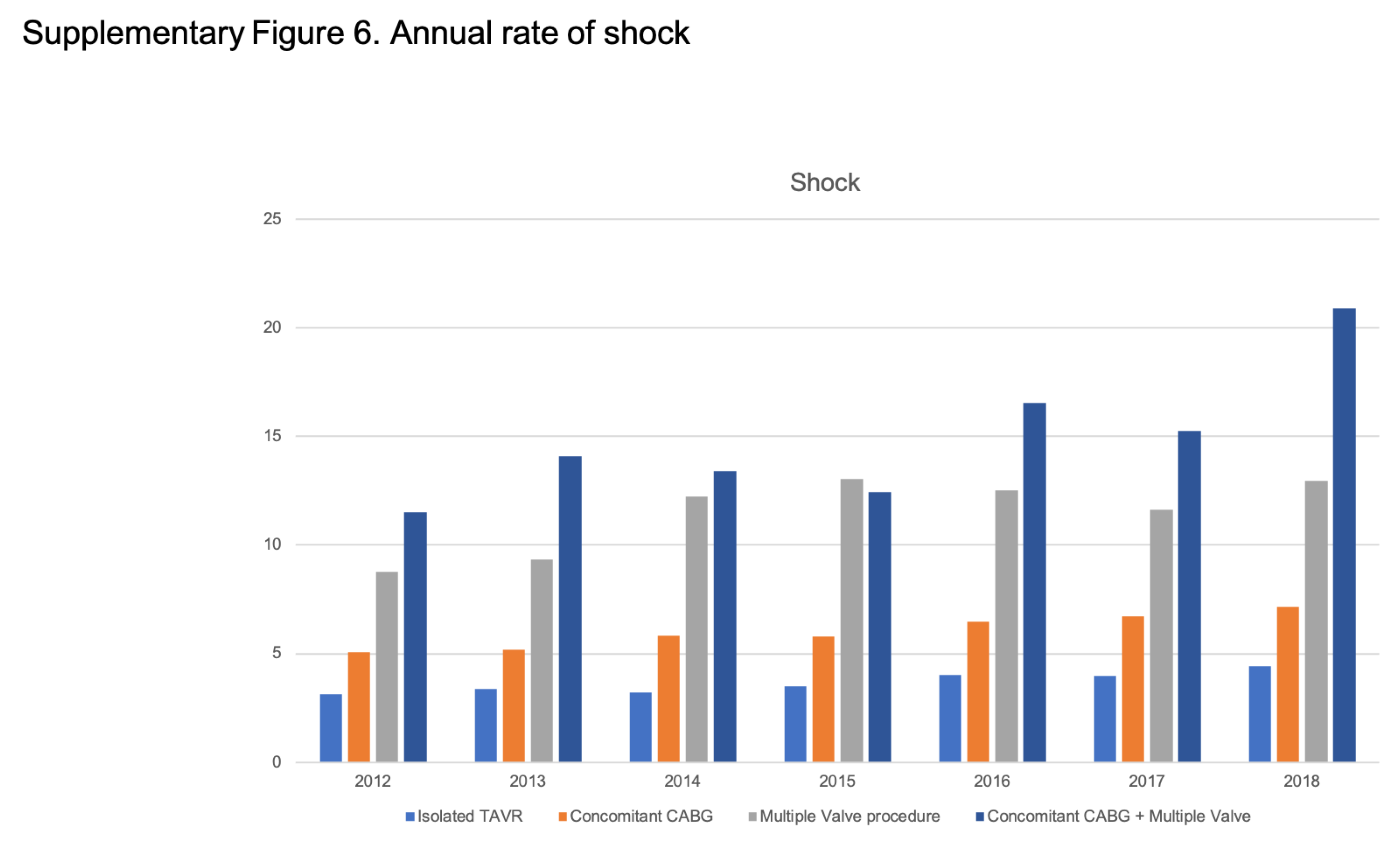

Supplement: Supplementary file 6 [file Image_6.TIF]

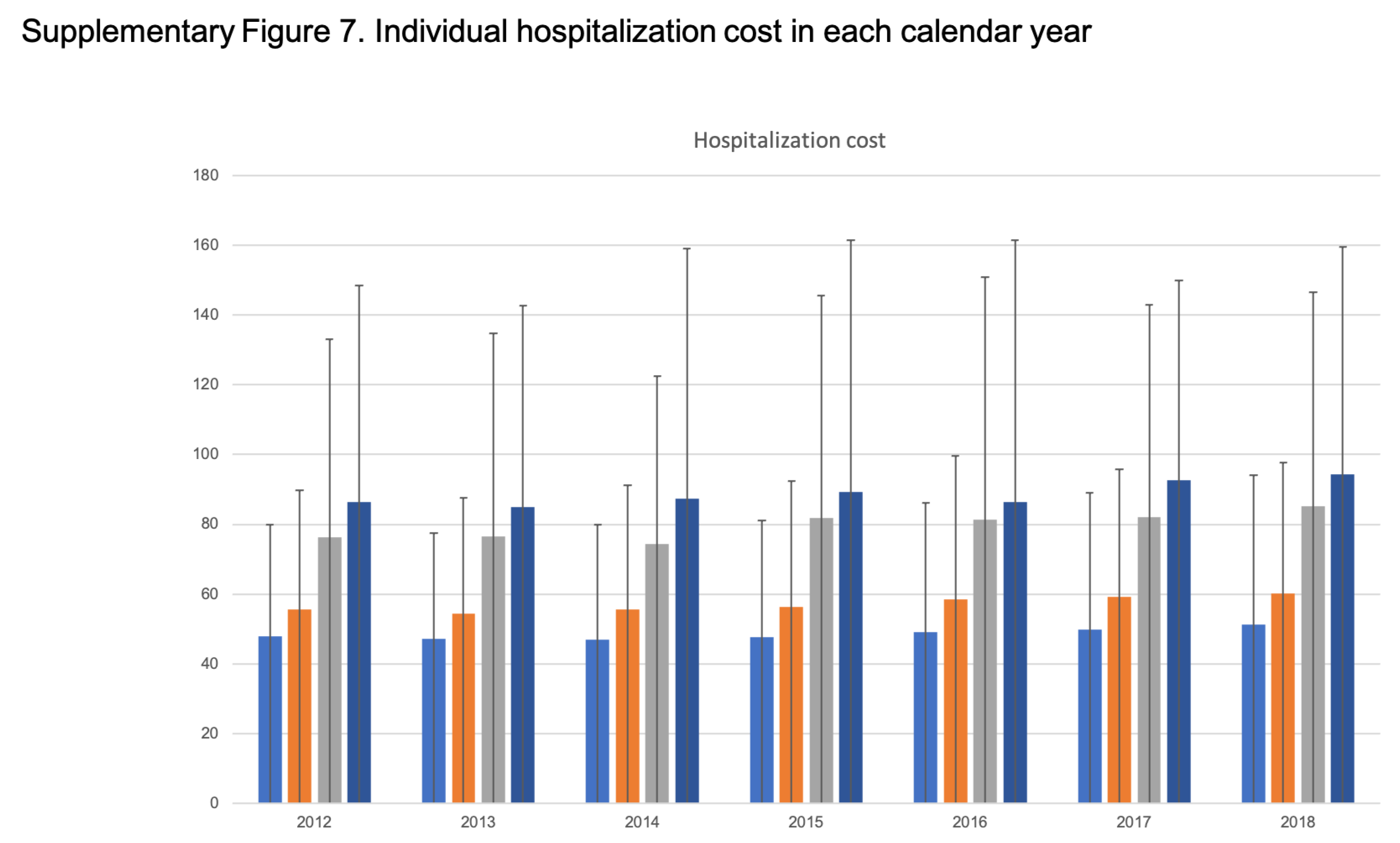

Supplement: Supplementary file 7 [file Image_7.TIF]
