# Supplementary material for: Trend and Impact of Concomitant CABG and Multiple-Valve Procedure on In-hospital Outcomes of SAVR Patients
Source: Front Cardiovasc Med. 2021 Sep 3;8:740084. doi: 10.3389/fcvm.2021.740084 (PMC8446624; doi:10.3389/fcvm.2021.740084)
Supplement: Supplementary file 8 [file Table_1.DOCX]

|  | | ICD 9 CODE | ICD 10 CODE |
| --- | --- | --- | --- |
| TAVR |  | 35.05, 35.06 | 02RF37H, 02RF38H, 02RF3JH, 02RF3KH, 02RF37Z, 02RF38Z, 02RF3JZ, 02RF3KZ |
| SAVR |  | 35.21, 35.22 | 02RF07Z, 02RF08Z, 02RF0KZ 02RF47Z, 02RF48Z, 02RF4JZ, 02RF4KZ, 02RF0JZ |
| AF |  | 427.31 | I48, I48.0, I48.1, I48.11, I48.19,  I48.2, I48.20, I48.21, I48.91 |
| Aortic stenosis |  | 395.0, 395.2, 396.0, 396.2, 424.1, 746.3 | [I06.0](https://www.icd10data.com/ICD10CM/Codes/I00-I99/I05-I09/I06-/I06.0), [I06.2,](https://www.icd10data.com/ICD10CM/Codes/I00-I99/I05-I09/I06-/I06.0)  [I08.0](https://www.icd10data.com/ICD10CM/Codes/I00-I99/I05-I09/I08-/I08.0), I35.0, I35.2, |
| CABG |  | 36.10, 36.11, 36.12, 36.13, 36.14,  36.15, 36.16, 39.61, 39.66 | CCS-44 |
| Mitral valve surgery |  | 35.23, 35.24 | 02UG07Z, 02NG0ZZ, 027G04Z, 02QG0ZZ, 02UG08Z, 02UG0JZ, 02UG0KZ, 025G0ZZ, 027G0DZ, 027G0ZZ, 02BG0ZX, 02BG0ZZ, 02RG07Z, 02RG08Z, 02RG0JZ, 02RG0KZ, 02VG0ZZ, 02CG0ZZ, 02WG07Z, 02WG08Z, 02WG0JZ, 02WG0KZ |
| Pulmonary valve surgery |  | 35.25, 35.26 | 02WH0KZ, 02WH07Z, 02WH0JZ, 027H04Z, 02RH08Z, 02TH0ZZ, 02RH0JZ, 02RH07Z, 02BH0ZZ, 02BH0ZX, 027H0ZZ, 027H0DZ, 025H0ZZ, 02UH0KZ, 02UH0JZ, 02UH08Z, 02UH07Z, 02QH0ZZ, 02NH0ZZ) |
| Tricuspid valve surgery |  | 35.27,35.28 | 02UJ0KZ, 027J04Z, 02NJ0ZZ, 02QJ0ZZ, 02UJ07Z, 02UJ08Z, 02UJ0JZ, 027J0DZ, 027J0ZZ, 02BJ0ZX, 02BJ0ZZ, 02RJ07Z, 02RJ0JZ, 02CJ0ZZ, 027J04Z, 02RJ08Z, 02RJ0KZ, 02WJ08Z, 02WJ0JZ, 02WJ07Z, 02WJ0KZ |
| Blood transfusion | | 99.01-99.09 | [30243N0](http://www.icd10data.com/ICD10PCS/Codes/3/0/2/4/30243N0), [30243N1](http://www.icd10data.com/ICD10PCS/Codes/3/0/2/4/30243N1), [30243P0](http://www.icd10data.com/ICD10PCS/Codes/3/0/2/4/30243P0), [30243P1](http://www.icd10data.com/ICD10PCS/Codes/3/0/2/4/30243P1), [30243H0](http://www.icd10data.com/ICD10PCS/Codes/3/0/2/4/30243H0), [30243H1](http://www.icd10data.com/ICD10PCS/Codes/3/0/2/4/30243H1), [30240N0](http://www.icd10data.com/ICD10PCS/Codes/3/0/2/4/30240N0), [30240N1](http://www.icd10data.com/ICD10PCS/Codes/3/0/2/4/30240N1), [30240P0](http://www.icd10data.com/ICD10PCS/Codes/3/0/2/4/30240P0), [30240P1](http://www.icd10data.com/ICD10PCS/Codes/3/0/2/4/30240P1), [30240H0](http://www.icd10data.com/ICD10PCS/Codes/3/0/2/4/30240H0), [30240H1](http://www.icd10data.com/ICD10PCS/Codes/3/0/2/4/30240H1), [30230H0](http://www.icd10data.com/ICD10PCS/Codes/3/0/2/3/30230H0), [30230H1](http://www.icd10data.com/ICD10PCS/Codes/3/0/2/3/30230H1), [30230N0](http://www.icd10data.com/ICD10PCS/Codes/3/0/2/3/30230N0), [30230N1](http://www.icd10data.com/ICD10PCS/Codes/3/0/2/3/30230N1), [30230P0](http://www.icd10data.com/ICD10PCS/Codes/3/0/2/3/30230P0), [30230P1](http://www.icd10data.com/ICD10PCS/Codes/3/0/2/3/30230P1), [30233N0](http://www.icd10data.com/ICD10PCS/Codes/3/0/2/3/30233N0), [30233N1](http://www.icd10data.com/ICD10PCS/Codes/3/0/2/3/30233N1), [30233P0](http://www.icd10data.com/ICD10PCS/Codes/3/0/2/3/30233P0),  [30233P1](http://www.icd10data.com/ICD10PCS/Codes/3/0/2/3/30233P1) |
| Acute stroke | | CCS-109 | CCS-CIR0220, CIR021,CIR024 |
| Acute kidney injury | | 584 | N170, N171, N172, N178, N179, N19, N990, R34, R944 |
| Hemodialysis | | 39.95, 585.6 | 5A1D00Z, 5A1D60Z |
| Cardiac complication | | 997.1, 42.30, 42.33, 370 | T8201XA, T8202XA, T8203XA,  T8209XA, T82222A, T82223A,  T82228A, T82867A, T82897A,  T82897A, I97710, I97790, I9788,  I9789, I9781, I9782 |
| Cardiac tamponade | | 423.3 | I314 |
| Cardiac arrest | | 427.5 | I46, I46.9, I46.2, I97.12, I97.71, I97.710, I97.711, I97.121 |
| Cardiogenic shock | | 785.51 | R570 |
| Permanent pacemaker | | 37.80, 37.83 | 02HK3JZ, 02H63JZ, 02HN0JZ, [02H60JZ](http://www.icd10data.com/ICD10PCS/Codes/0/2/H/6/02H60JZ), [02H60NZ](http://www.icd10data.com/ICD10PCS/Codes/0/2/H/6/02H60NZ), [02H63JZ](http://www.icd10data.com/ICD10PCS/Codes/0/2/H/6/02H63JZ), [02H63NZ](http://www.icd10data.com/ICD10PCS/Codes/0/2/H/6/02H63NZ), [02H64JZ](http://www.icd10data.com/ICD10PCS/Codes/0/2/H/6/02H64JZ), [02H64NZ](http://www.icd10data.com/ICD10PCS/Codes/0/2/H/6/02H64NZ), [02HK0JZ](http://www.icd10data.com/ICD10PCS/Codes/0/2/H/K/02HK0JZ), [02HK0NZ](http://www.icd10data.com/ICD10PCS/Codes/0/2/H/K/02HK0NZ), [02HK3JZ](http://www.icd10data.com/ICD10PCS/Codes/0/2/H/K/02HK3JZ), [02HK3NZ](http://www.icd10data.com/ICD10PCS/Codes/0/2/H/K/02HK3NZ), [02HK4JZ](http://www.icd10data.com/ICD10PCS/Codes/0/2/H/K/02HK4JZ), [02HK4NZ](http://www.icd10data.com/ICD10PCS/Codes/0/2/H/K/02HK4NZ), 02HN4JZ, 0JH604Z, 0JH634Z, 0JH605Z, 0JH607Z, 0JH635Z, [0JH606](http://www.icd10data.com/ICD10PCS/Codes/0/J/H/6/0JH606Z)Z, 0JH634Z, 0JH635Z, 0JH636Z, 0JH637Z |
| Acquired pneumonia | | 486, 481, 482.8, 482.3 | J13, J14, J15.1, J15.2, J15.4, J15.5, J15.6, J15.7, J15.8, J15.9, J16.0 |
| Sepsis | | 038, 995.91, 995.92, 996.64,  999.31, 999.32 | A410, A411, A412, A413, A414,  A415, A418, A419, A400, A401,  R6520, T8351XA, T80219A,  T80211A, A403, A408, A409,  B377, R651, T835, T857,  T827, T814 |
| Mechanical ventilation | | 96.72 | 5A1955Z |
| Tracheostomy | | 311, 31.21, 31.29 | 0B110F4, 0B110Z4, 0B113F4, 0B113Z4  0B114F4, 0B114Z4 |
| Gastrostomy | | 43.11, 43.19 | 0DH63UZ, 0DH64UZ, 0D16074,  0D160J4, 0D160K4, 0D160Z4,  0D163J4, 0D16474, 0D164J4,  0D164K4, 0D164Z4, 0D16874  0D168J4, 0D168K4, 0D168Z4  0DH60UZ |

**Supplementary table 1**: List of the used ICD-9. ICD-10, and CCS codes. AF = atrial fibrillation; AVR = aortic valve replacement; CABG = coronary artery bypass grafting; SAVR = surgical aortic valve replacement; TAVR = transcatheter aortic valve replacement.
