# Supplementary material for: Trend and Impact of Concomitant CABG and Multiple-Valve Procedure on In-hospital Outcomes of SAVR Patients
Source: Front Cardiovasc Med. 2021 Sep 3;8:740084. doi: 10.3389/fcvm.2021.740084 (PMC8446624; doi:10.3389/fcvm.2021.740084)
Supplement: Supplementary file 9 [file Table_2.DOCX]

Supplementary table 2: Baseline characteristics for SAVR with or without concomitant CABG in the matched cohorts

|  |  |  | | |
| --- | --- | --- | --- | --- |
|  | Isolated  SAVR  (n = 25,473) | | Concomitant  CABG  (n =25,473) | P Value |
| Age, yrs | 72.1 ± 8.9 | | 72.1± 8.5 | 0.76 |
| Female | 7,187(28.2) | | 7,214 (28.3) | 0.80 |
| Hypertension | 18,439 (72.4) | | 18,617 (73.1) | 0.08 |
| Diabetes | 6,420 (25.2) | | 6,647 (26.1) | 0.02 |
| Diabetes with chronic complications | 3,224 (12.7) | | 2,906 (11.4) | <0.001 |
| Chronic lung disease | 5,601 (22.0) | | 5,559 (21.8) | 0.66 |
| Congestive heart failure | 450 (1.8) | | 429 (1.7) | 0.50 |
| Atrial fibrillation | 12,254 (48.1) | | 12,218 (48.0) | 0.76 |
| Chronic renal disease | 4,784 (18.8) | | 4,763 (18.7) | 0.82 |
| Anemia | 4,254 (16.7) | | 4,763 (16.6) | 0.74 |
| Arthritis | 723 (2.8) | | 708 (2.8) | 0.71 |
| Coagulopathy | 9,378 (36.8) | | 9,328 (36.6) | 0.65 |
| Hypothyroidism | 3,301 (13.0) | | 3,309 (13.0) | 0.93 |
| Liver disease | 473 (1.9) | | 467 (1.8) | 0.87 |
| Obesity | 5,835 (22.9) | | 5,741 (22.5) | 0.33 |
| Weight loss | 1,195 (4.7) | | 1,181 (4.7) | 0.78 |
| Peripheral vascular disease | 4,560 (17.9) | | 4,628 (18.2) | 0.44 |
| Pulmonary circulation disorder | 101 (0.4) | | 106 (0.4) | 0.78 |
| Tumor | 327 (1.3) | | 325 (1.3) | 0.97 |
| Teaching hospital | 19,438 (76.3) | | 19,388 (76.1) | 0.61 |
| Rural location | 660 (2.6) | | 649 (2.6) | 0.78 |
| Large hospital bed size | 17,366 (68.2) | | 17,368 (68.2) | 0.99 |
| Primary payer |  | |  |  |
| Medicare/Medicaid | 19,813 (77.8) | | 19,804 (77.7) | 0.83 |
| Private insurance | 4,908 (19.3) | | 4877 (19.1) | 0.83 |
| Elective admission | 18,135 (71.2) | | 17,914 (70.3) | 0.03 |
|  |  | |  |  |

Values are count (percent), mean ± SD. SAVR = surgical aortic valve replacement; CABG = coronary artery bypass grafting.
