# Supplementary material for: Trend and Impact of Concomitant CABG and Multiple-Valve Procedure on In-hospital Outcomes of SAVR Patients
Source: Front Cardiovasc Med. 2021 Sep 3;8:740084. doi: 10.3389/fcvm.2021.740084 (PMC8446624; doi:10.3389/fcvm.2021.740084)
Supplement: Supplementary file 10 [file Table_3.DOCX]

Supplementary table 3: Baseline Characteristics for SAVR with or without Multiple Valves Procedure in Matched Cohorts

|  |  |  | | |
| --- | --- | --- | --- | --- |
|  | Isolated  SAVR  (n = 5,589) | | Multiple valves  Procedure  (n =5,589) | P Value |
| Age, yrs | 69.3 ± 9.6 | | 69.0± 9.4 | 0.07 |
| Female | 2,617 (46.8) | | 2,640 (47.2) | 0.67 |
| Hypertension | 3,196 (57.2) | | 3,184 (57.0) | 0.83 |
| Diabetes | 876 (15.7) | | 889 (15.9) | 0.76 |
| Diabetes with chronic complications | 612 (11.0) | | 605 (10.8) | 0.86 |
| Chronic lung disease | 1,395 (25.0) | | 1,362 (24.4) | 0.48 |
| Congestive heart failure | 288 (5.2) | | 315 (5.6) | 0.28 |
| Atrial fibrillation | 3,628 (64.9) | | 3,543 (63.4) | 0.10 |
| Chronic renal disease | 1,342 (24.0) | | 1,302 (23.3) | 0.41 |
| Anemia | 1,216 (21.8) | | 1,164 (20.8) | 0.24 |
| Arthritis | 239 (4.3) | | 222 (3.9) | 0.39 |
| Coagulopathy | 2,558 (45.8) | | 2,548 (45.6) | 0.86 |
| Hypothyroidism | 806 (14.4) | | 843 (15.1) | 0.34 |
| Liver disease | 207 (3.7) | | 229 (4.1) | 0.30 |
| Obesity | 1,099 (19.7) | | 1,090 (19.5) | 0.85 |
| Weight loss | 597 (10.7) | | 622 (11.1) | 0.47 |
| Peripheral vascular disease | 821 (14.7) | | 829 (14.8) | 0.85 |
| Pulmonary circulation disorder | 65 (0.1) | | 71 (0.1) | 0.67 |
| Tumor | 61 (1.1) | | 67 (1.1) | 0.66 |
| Teaching hospital | 4,663 (83.4) | | 4,671 (83.6) | 0.76 |
| Rural location | 81 (1.4) | | 72 (1.3) | 0.76 |
| Large hospital bed size | 4,084 (73.1) | | 4,111 (73.6) | 0.19 |
| Primary payer |  | |  |  |
| Medicare/Medicaid | 4,018 (71.9) | | 4,083 (73.1) | 0.18 |
| Private insurance | 1,366 (24.4) | | 1,278 (22.9) | 0.05 |
| Elective admission | 3,553 (63.6) | | 3,541 (63.4) | 0.83 |
|  |  | |  |  |

Values are count (percent), mean ± SD. SAVR = surgical aortic valve replacement.
