# Supplementary material for: Trend and Impact of Concomitant CABG and Multiple-Valve Procedure on In-hospital Outcomes of SAVR Patients
Source: Front Cardiovasc Med. 2021 Sep 3;8:740084. doi: 10.3389/fcvm.2021.740084 (PMC8446624; doi:10.3389/fcvm.2021.740084)
Supplement: Supplementary file 11 [file Table_4.DOCX]

Supplementary table 4: Baseline Characteristics for isolated SAVR and Concomitant CABG + Multiple Valves Procedure in Matched Cohorts

|  |  |  | | |
| --- | --- | --- | --- | --- |
|  | Isolated  SAVR  (n = 2,286) | | Concomitant  CABG + Multiple Valves Procedure  (n =2,286) | P Value |
| Age, yrs | 72.1 ± 9.4 | | 71.8± 8.8 | 0.33 |
| Female | 856 (37.4) | | 850 (37.2) | 0.88 |
| Hypertension | 1,324 (57.9) | | 1,372 (60.0) | 0.16 |
| Diabetes | 437 (25.2) | | 447 (26.1) | 0.18 |
| Diabetes with chronic complications | 321 (14.0) | | 331 (14.5) | 0.70 |
| Chronic lung disease | 552 (24.1) | | 564 (24.7) | 0.70 |
| Congestive heart failure | 152 (6.6) | | 142 (6.2) | 0.59 |
| Atrial fibrillation | 1,341 (58.7) | | 1,373 (60.1) | 0.76 |
| Chronic renal disease | 630 (27.6) | | 642 (28.1) | 0.72 |
| Anemia | 384 (16.8) | | 415 (18.2) | 0.24 |
| Arthritis | 75 (3.3) | | 70 (3.1) | 0.74 |
| Coagulopathy | 1,076 (47.1) | | 1,077 (47.1) | 0.99 |
| Hypothyroidism | 363 (15.9) | | 339 (14.8) | 0.35 |
| Liver disease | 62 (2.7) | | 70 (3.1) | 0.54 |
| Obesity | 378 (16.5) | | 379 (16.6) | 0.99 |
| Weight loss | 240 (10.5) | | 256 (11.2) | 0.48 |
| Peripheral vascular disease | 410 (17.9) | | 444 (19.4) | 0.21 |
| Pulmonary circulation disorder | 49 (2.1) | | 35 (1.5) | 0.15 |
| Tumor | 24 (1.0) | | 25 (1.0) | 0.68 |
| Teaching hospital | 1,849 (80.9) | | 1,855 (81.1) | 0.62 |
| Rural location | 45 (2.0) | | 37 (1.6) | 0.05 |
| Large hospital bed size | 1,566 (68.5) | | 1,584 (69.3) | 0.72 |
| Primary payer |  | |  |  |
| Medicare/Medicaid | 1,826 (79.9) | | 1,844 (80.7) | 0.14 |
| Private insurance | 406 (17.8) | | 367 (16.1) | 0.13 |
| Elective admission | 1,400 (61.2) | | 1,279 (60.7) | 0.72 |
|  |  | |  |  |

Values are count (percent), mean ± SD. SAVR = surgical aortic valve replacement; CABG = coronary artery bypass grafting.
